# Retraction: Hyperglycemia via activation of thromboxane A2 receptor impairs the integrity and function of blood-brain barrier in microvascular endothelial cells

**DOI:** 10.18632/oncotarget.28675

**Published:** 2024-11-22

**Authors:** Zhihong Zhao, Jue Hu, Xiaoping Gao, Hui Liang, Haiya Yu, Suosi Liu, Zhan Liu

**Affiliations:** ^1^Department of Neurology, The First Affiliated Hospital (People’s Hospital of Hunan Province), Hunan Normal University, Changsha, Hunan, China; ^2^Department of Neurology, Changsha Central Hospital, Changsha, Hunan, China; ^3^Department of Neurology, The People’s Hospital of Xishui, Huangang, Hubei, China; ^4^Department of Clinical Nutrition and Gastroenterology, The First Affiliated Hospital (People’s Hospital of Hunan Province), Hunan Normal University, Changsha, Hunan, China; ^*^These authors contributed equally to this work

**This article has been retracted:** The authors contacted Oncotarget, stating, ‘In this paper, we occasionally reused some western blot bands for other papers. For multiple errors in our paper, we want to retract our paper published in your journal.’ Oncotarget requested an investigation by the authors’ institution (The First Affiliated Hospital (People’s Hospital of Hunan Province), Hunan Normal University, Changsha, Hunan, China) but we received no reply. Oncotarget conducted an internal image forensics report that revealed several instances of overlap between figures from previously published unrelated papers [1–3] and a concurrently published paper [4]. Specifically, Figure 6C duplicates Figure 4A of [1]; Figure 1C duplicates Figure 1E of the paper, which has been retracted [2]; Figure 6, panels A and B, duplicates images in Figure 3A of [3]; Figure 1, panels A and B, duplicates Figure 1, panels A and B, of another paper [4]; Figure 2, panels A and B, duplicates Figure 2, panels A and B of [4]; Figure 3, panels A, B and C, duplicates Figure 3, panels A, B and C, of [4]; Figure 5, panels A, B and C, duplicates Figure 4, panels A, B and C of [4]. As a result, the Editorial decision was made to retract this paper. All authors agreed with the decision.

Original article: Oncotarget. 2017; 8:30030–30038. 30030-30038. https://doi.org/10.18632/oncotarget.16273
